# Society-oriented AI governance: a parallel layered model and multi-actor coordination framework

**DOI:** 10.3389/frai.2026.1872620

**Published:** 2026-07-03

**Authors:** Aditya Firman Ihsan, Thomhert Suprapto Siadari, Andry Alamsyah, Kemas Muslim Lhaksmana, Helni Mutiarsih Jumhur

**Affiliations:** 1 School of Computing, Telkom University, Bandung, Indonesia; 2 School of Electrical Engineering, Telkom University, Bandung, Indonesia; 3 School of Economics and Business, Telkom University, Bandung, Indonesia

**Keywords:** federated governance, large language models, layered governance model, multi-actor framework, social layer, society-oriented governance

## Abstract

The rapid and unrestricted public adoption of large language models and other openly accessible AI technologies has exposed a fundamental structural weakness in existing AI governance frameworks: the systematic underrepresentation of society as the ultimate recipient of AI’s consequences. Most current frameworks treat societal impact as a downstream consideration rather than a foundational design criterion. This paper proposes society-oriented governance — the principle that societal reception and utilization of AI must serve as the primary design criterion for governance frameworks — as a structural response to this failure.To operationalize this principle, we develop two interconnected analytical frameworks: a governance development framework that replaces the sequential, hierarchical structure of a prior layered governance model with a parallel and dynamic model in which the technical, ethical, and social layers are developed simultaneously and in continuous mutual interaction; and a multiple-actors framework that maps stakeholders across the full lifecycle of AI utilization and proposes coordination mechanisms suited to both centralized and federated governance paradigms. A key finding is the structural distinction between the 4-actors model, applicable to professionally mediated AI deployment, and the 3-actors model, which describes the dominant pattern of openly accessible consumer AI where no professional intermediary exists. This distinction reveals the social layer as the most critical and most underdeveloped layer in current AI governance, and motivates concrete recommendations for strengthening it through socially responsive design standards, national-level regulatory obligations, and federated multi-stakeholder coordination. These contributions are grounded in AI governance theory — in governance design, institutional architecture, and stakeholder coordination — and are not proposed as a sociological theory of society.

## 1 Introduction

Artificial intelligence (AI) has become a defining feature of contemporary society, reshaping industries, public services, and everyday human interaction at an unprecedented pace. Yet the governance of AI has struggled to keep up. Despite the proliferation of ethical guidelines, regulatory proposals, and governance frameworks, a persistent gap remains between the abstract principles articulated in policy documents and their operationalization in practice (Birkstedt et al., 2023; Krijger, 2021; Morley et al., 2023). This gap is not merely technical — it reflects a deeper structural problem: existing AI governance frameworks are predominantly designed around the concerns of developers and institutions, with society as the ultimate recipient of AI’s consequences remaining underrepresented in governance design (Coeckelbergh, 2024; Kieslich et al., 2022; Stix, 2021; Wittmann and Meynhardt, 2025). This structural weakness is visible across the five most internationally influential AI governance frameworks, examined in detail in Section 2.

Current governance approaches tend to treat societal impact as a downstream consideration — something to be addressed after technical development and ethical deliberation have already taken place. This sequencing, implicit in influential layered models such as that of Gasser and Almeida (2017), assumes that governance can be developed hierarchically and top-down, from technical standards through ethical principles to social norms. However, empirical realities — most starkly illustrated by the rapid, unrestricted public adoption of large language models such as ChatGPT — demonstrate that societal impact neither waits for nor follows neatly from upstream governance decisions. Harms materialize at the point of public use, often before regulatory frameworks have had the opportunity to respond (Taeihagh et al., 2021). A governance model that positions society at the end of a linear chain is therefore structurally ill-suited to address the challenges of openly accessible AI.

This paper proposes a fundamental reorientation: AI governance must be designed with society not as a passive endpoint, but as its core organizing purpose. We term this the principle of society-oriented AI governance. While variants of ‘society-oriented’ or ‘society-centered’ framing appear in adjacent literature — in decision-support modeling (Yang et al., 2005), in responsible innovation discourse (Stilgoe et al., 2013), and in human-centered AI design (Shneiderman, 2022) — our use is distinct. It is also distinct from two closely related recent contributions. Coeckelbergh (2024) diagnoses a “democracy deficit” in AI governance and argues for a more active role of citizens and end-users; our framework shares this concern but operationalizes it structurally — as a governance design criterion governing how layers are built and who bears accountability at each stage, not only as a normative call for democratic participation. Sigfrids et al. (2023) propose expanding human-centered AI governance from user-centered to community- and society-centered perspectives, which aligns with our social layer emphasis; our contribution adds the parallel-layer architecture and the 3-actors model as concrete mechanisms for achieving that expansion.

In all cases, our use of “society-oriented” denotes a governance design principle that positions societal reception and utilization as the foundational criterion from which governance architecture is derived, rather than as a values commitment, a stakeholder consideration, or a design quality. This distinction also applies to adjacent concepts that may appear superficially similar. Human-centered AI (Norman, 2023; Shneiderman, 2022) positions the individual user’s needs and cognitive experience as the primary design reference; society-oriented governance positions the collective conditions of societal reception — including communities, institutions, and populations not yet using a system — as the foundational design criterion. Citizen-centered governance and participatory governance frameworks emphasize procedural inclusion of citizens in decision-making processes; our framework goes further by treating societal reception structurally, as the criterion from which governance architecture is derived, not merely as a value to be procedurally honored. Socio-technical governance approaches recognize the co-constitution of technical systems and social structures; our contribution adds a concrete architectural mechanism — the parallel-layer model and the 3-actors framework — for operationalizing that recognition in governance design. In short, society-oriented governance is not a reframing of existing concerns but a structural reorientation with specific architectural implications. This reorientation is proposed as a governance design principle, not a sociological or normative theory of society. Also, this has direct implications for how governance layers interact, how responsibilities are distributed among stakeholders, and how global standards are reconciled with local contextual conditions.

To implement this principle, we propose two interconnected analytical frameworks. First, a governance development framework that modifies the layered model of Gasser and Almeida (2017) by replacing its hierarchical, sequential structure with a parallel and dynamic model in which the technical, ethical, and social layers are developed simultaneously and in continuous mutual interaction. Second, a multiple-actor framework that maps the stakeholders involved across the full lifecycle of AI utilization — from research and innovation to public use — and proposes coordination mechanisms suited to both centralized and federated governance paradigms. Where the governance development framework establishes what needs to be governed at each layer, the multiple-actor framework addresses who governs it and how coordination is sustained across sectors and scales. Together, these frameworks provide a structured analytical lens for diagnosing governance failures and designing context-sensitive responses grounded in the society-oriented principle.

The remainder of this paper is structured as follows. Section 2 examines the societal implications of openly accessible AI technologies, establishes the methodological approach, and reviews existing major governance frameworks. Sections 3 and 4 develop the two proposed frameworks in detail. Section 5 discusses practical implications, including federated governance coordination, the urgency of strengthening social layer governance, and the division of responsibilities across global and national scopes. Section 6 concludes with recommendations for future research and implementation.

## 2 AI toward society

### 2.1 Lesson from ChatGPT and the rise of openly accessible AI

The emergence of large language models (LLMs) as publicly accessible technologies marks a critical inflection point in the relationship between AI and society. Unlike previous generations of AI systems that were largely confined to specialized professional or institutional settings, LLMs such as ChatGPT, Gemini, Claude, and open-source variants like Llama and Mistral are designed for general-purpose use by anyone with an internet connection, regardless of age, background, or expertise (Bommasani et al., 2023). This shift from controlled, domain-specific deployment to open, unrestricted public access represents not merely technological advancement but a fundamental change in the governance landscape.

A particularly important characteristic of generative AI systems — one that compounds the governance challenges described in this paper — is their inherently general-purpose nature. Unlike previous technologies designed around relatively constrained use cases, systems such as ChatGPT are not built for a defined set of applications; their functional scope is determined by user intent at the point of interaction (Amankwah-Amoah et al., 2024). This versatility makes it structurally impossible to fully anticipate the range of deployment contexts, user populations, or emergent harms in advance — which is precisely why pre-deployment societal impact assessment and ongoing societal feedback mechanisms, rather than application-specific regulation alone, are necessary governance instruments.

ChatGPT, since its public release in November 2022, has become the most prominent example of this shift. In healthcare, it has shown promise for literature review, clinical decision support, and drug discovery (Sallam, 2023). In academia, it has challenged existing frameworks for research integrity, authorship, and scholarly publishing (Lund et al., 2023; Mijwil et al., 2023). In commerce, media, and everyday life, it has reshaped how millions of people access information, produce content, and interact with institutions (Mattas, 2023). However, ChatGPT is best understood not as an isolated phenomenon but as the most visible instance of a broader class of AI systems whose defining characteristic is open, unrestricted societal reach.

The governance vulnerabilities exposed by ChatGPT’s adoption are therefore not specific to that product — they are structural features of openly accessible AI as a category. These systems are accessible to anyone regardless of age, background, educational level, or intent, effectively bypassing the staged, professional-mediated governance pathways that most existing frameworks presuppose (Ray and Das, 2023). Concerns about academic dishonesty, epistemic manipulation, labor displacement, and the erosion of critical thinking capacities have emerged faster than regulatory responses (Bommasani et al., 2023). Critically, these are not technical failures — they are governance failures, rooted in the structural absence of an adequate social layer to mediate between AI deployment and public use.

To illustrate the diagnostic utility of this framing, consider three concrete failure patterns observable in ChatGPT’s public adoption. First, the rapid uptake among minors without age-sensitive access controls represents a social layer failure: no pre-deployment mechanism existed to calibrate access to the actual user population. Second, the spread of AI-assisted academic dishonesty across educational institutions reflects the absence of usage transparency mechanisms and institutional oversight standards — instruments that should have been embedded at the platform level before public release. Third, documented cases of users in psychological distress receiving unmodulated responses from the system point to the absence of mandatory community impact assessment and incident reporting obligations. In each case, the failure is not attributable to a technical flaw in the underlying model, nor to an absence of ethical principles in its developers’ stated commitments — it is attributable to the structural absence of social layer governance at the point of public deployment. This pattern of failures is what the frameworks proposed in this paper are designed to diagnose and address.

This pattern extends well beyond LLMs. Deep fake applications, AI-generated artwork platforms, algorithmic hiring tools, and autonomous recommendation systems all share the same structural characteristic: they reach society directly, without a professional intermediary who can exercise contextual judgment, apply domain ethics, or bear institutional accountability for outcomes. As Bommasani et al. (2023) observe, foundation models have a social footprint that extends far beyond what any single actor can anticipate or govern. In the years ahead, increasingly capable AI systems will reach public hands faster, in more domains, and with less gatekeeping — making governance frameworks oriented around the point of social use, not just technical development, an urgent and escalating priority.

Crucially, this governance failure cannot be attributed to an absence of frameworks. The NIST AI Risk Management Framework and the EU AI Act were in active development during ChatGPT’s global rise. Their structural limitation was orientation: both were designed to govern AI at the point of development and organizational deployment, not at the point of unrestricted public use.

### 2.2 Methodological approach

This paper employs conceptual analysis grounded in the AI governance literature. The approach proceeds in three steps, each directly motivated by the societal challenge established in Section 2.1.

First, we conduct a structured comparative analysis of the five most internationally prominent AI governance frameworks, applying a common diagnostic question: does each framework treat societal reception and utilization as a foundational design criterion, or as a downstream compliance outcome? This analysis, presented in Section 2.3, establishes empirical motivation for the society-oriented reorientation proposed in the following sections.

Second, we develop two interconnected analytical frameworks through conceptual extension and modification. The governance development framework builds on and departs from the layered model of Gasser and Almeida (2017), replacing its sequential structure with a parallel and dynamic one. The multiple-actor framework generalizes a domain-specific healthcare governance model (Siadari et al., 2022) into a cross-sectoral architecture applicable across AI deployment contexts.

Third, the ChatGPT case is used throughout as a paradigmatic illustration — a concrete, well-documented instance of structurally ungoverned public AI adoption that reveals governance failures applicable to openly accessible AI as a category, not as a formal case study. Both frameworks produced are analytical lenses and strategic architectures, not implementation protocols; validation directions are discussed in the Conclusion.

### 2.3 Layered AI governance

**Figure 1:**
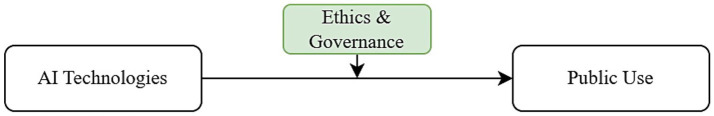
Main goals of society-oriented AI ethics and governance is to be a gate or passage that filters and directs how AI technology can be used productively for society.

As illustrated in Figure 1, governance functions as a filtering bridge between AI technologies and society, ensuring that public utilization occurs within a framework of accountability and control. Without effective governance, AI deployment produces disorganized conditions in which there is no coherent mechanism for directing use, preventing harm, or distributing responsibility. The ChatGPT case exemplifies this precisely: a technology of extraordinary capability became globally accessible without adequate social layer governance in place to manage its varied, unpredictable reception across vastly different communities, age groups, and cultural contexts. Crucially, governance is not a universal or static instrument — it must be sensitive to context, adaptive to change, and responsive to the social conditions in which AI is received.

The pathway from AI innovation to public use is a complex, multi-stage process involving diverse actors, institutions, and regulatory environments. Governing this pathway requires an analytical framework capable of capturing its full complexity without collapsing distinct stages into a single undifferentiated object of governance. For this purpose, we adopt and build upon the layered governance model proposed by Gasser and Almeida (2017), whose concept of modularity offers a productive starting point. By organizing the AI governance system into discrete but interrelated layers, the framework reduces analytical complexity while preserving the ability to diagnose layer-specific failures — a critical feature for the society-oriented approach developed in this paper.

Gasser and Almeida (2017) proposed a three-layer model consisting of: a technical layer (data governance, algorithmic accountability, standards), an ethical layer (criteria and principles), and a social layer (norms, regulation, legislation). In their model, these layers are arranged hierarchically and sequentially. As we discuss in the following section, we depart from this sequential structure while retaining the three-layer architecture, proposing instead a parallel and dynamic model in which all three layers must be developed simultaneously and in continuous interaction — a modification that better reflects the empirical realities of AI deployment and the demands of society-oriented governance.

The five most internationally influential AI governance frameworks — OECD AI Principles (2019, updated OECD, 2024), the UNESCO Recommendation on the Ethics of AI (UNESCO, 2021), the EU AI Act (European Parliament, & the Council of the European Union, 2024), the NIST AI Risk Management Framework (National Institute of Standards, & Technology, 2023), and ISO 23894 (ISO/IEC, 2023) — represent genuine advances in governance thinking, yet share a structural assumption that limits their adequacy. The OECD AI Principles invoke society as a beneficiary of values such as transparency, robustness, and accountability, but not as a criterion shaping how governance frameworks are designed. The UNESCO Recommendation calls on member states to assess societal impact and protect human rights, but its governance architecture centers on national institutions, not conditions of societal reception. The EU AI Act assigns risk categories to AI applications and imposes obligations on providers and deployers; societal reception is a compliance outcome, not a design criterion. The NIST AI Risk Management Framework provides the most detailed organizational guidance, organizing risk management into Govern, Map, Measure, and Manage functions — yet society appears in the Govern function as a category of impact to be considered, not as an actor whose needs structure the framework. ISO 23894:2023 follows the same organization-centric logic. Across all five, societal reception of AI is treated as a downstream effect to be assessed and mitigated. Society-oriented governance proposes the inverse: governance frameworks should be designed from the perspective of how AI is received and utilized by society, with technical and ethical governance flowing from that foundation.

## 3 Governance development framework

### 3.1 Modified layered model

Building on the layered governance model introduced in Section 2, this section develops the governance development framework in full detail. We adopt the three-layer architecture of Gasser and Almeida (2017) — technical, ethical, and social — but substantially modify both the content of each layer and the structural relationships among them. Our core modification is to replace the original sequential, hierarchical model with a parallel and dynamic one: all three layers must be developed simultaneously and in continuous mutual interaction. This reflects a fundamental empirical reality of AI deployment — societal conditions change dynamically, ethical challenges emerge in response to social shifts, and technical systems must adapt accordingly. Governance that treats these as sequential steps will always lag behind the realities it is meant to govern.

The social layer encompasses two analytically distinct but interrelated domains: the informal normative environment (community norms, public expectations, cultural attitudes toward AI) and the formal regulatory environment (legislation, enforceable standards, institutional oversight mechanisms). It is important to distinguish the social layer from the ethical layer with which it interacts. The ethical layer operates at the level of principles and normative criteria — it translates moral values into actionable design and policy guidance. The social layer, by contrast, operates at the level of implementation and reception — it governs the conditions under which AI technologies actually reach and are used by the public. Digital literacy programs, access controls, participatory governance structures, and transparency requirements are social layer instruments not because they express ethical values, but because they function as governance mechanisms mediating the interface between AI systems and public use. This boundary clarification means that when an ethical principle such as transparency is translated into a mandatory disclosure requirement enforceable by law, it has migrated from the ethical layer into the social layer — a migration that the parallel-layer model explicitly requires and tracks.

The ethical layer provides the philosophical and normative foundation that connects technical development to social outcomes. It translates abstract moral principles — fairness, transparency, accountability, and human dignity — into actionable criteria that guide both the design of AI systems and the formulation of regulatory policy (Andrada et al., 2022; Vinuesa et al., 2020). Crucially, the ethical layer in our framework operates bidirectionally: it is responsive to conditions in the social layer and translates its outputs upward into the technical layer. This distinguishes our treatment from purely principle-based approaches, where ethics functions as a one-time design input rather than a continuous governance mechanism.

The technical layer encompasses the full infrastructure of AI research, development, and standardization: AI systems whether physical or cyber, data management practices, algorithmic accountability mechanisms, and the technical standards that govern their interaction (Gasser and Almeida, 2017). Of the three layers, the technical layer is currently the most developed in governance terms. However, technical governance alone is insufficient; without corresponding maturity in the ethical and social layers, well-governed technical development can still produce poorly governed societal outcomes.

**Figure 2:**
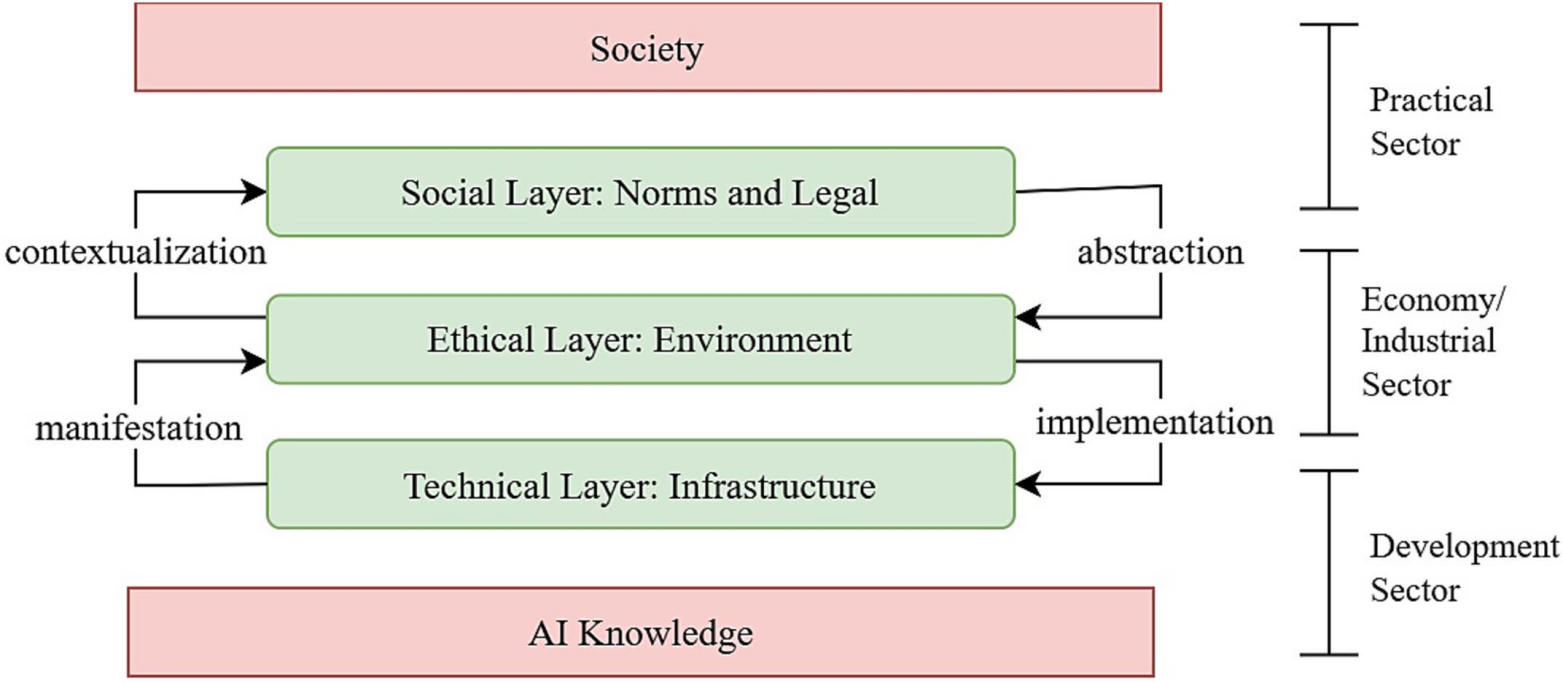
Proposed modification of layered models of AI Governance.

The key structural departure from Gasser and Almeida (2017) is that our model treats the three layers not as a sequential hierarchy but as a system of simultaneous, mutually constitutive processes, as depicted in Figure 2. The interaction between layers is bidirectional and continuous. New technical innovations should prompt immediate reconsideration of ethical principles and social layer readiness; equally, shifts in social conditions should flow upward to reshape ethical frameworks and inform technical development priorities. Two illustrative cases demonstrate this dynamic. In autonomous vehicle development, technical design choices must be guided by ethical principles of human safety, and deployment must account for social-layer realities including misuse risks. In healthcare AI, government regulations requiring transparency (social layer) are informed by patient autonomy principles (ethical layer) and require technical compliance through auditable algorithms (technical layer). In both cases, the interaction is iterative, reciprocal, and dynamic — making parallel, synchronized development not merely desirable but necessary.

This transition from sequential to parallel governance development has a productive analogy in software engineering. The shift from traditional V-model development — in which requirements, design, implementation, and validation proceed in strict sequence — toward Agile methodologies (Abrahamsson et al., 2010) reflects a similar recognition that iterative, concurrent processes produce more adaptive and resilient outcomes than linear ones. Just as Agile development replaced the assumption that requirements can be fully specified upfront with continuous iteration between development and feedback, the parallel-layer model replaces the assumption that governance can be developed hierarchically with continuous interaction across all three layers simultaneously.

It is worth distinguishing the present framework from the four-layered AI governance model proposed by Wirtz et al. (2022), which organizes governance around six risk-and-guideline categories (technological, data-analytical, informational, economic, social, and ethical-legal). That framework is a *risk-classification* architecture: it maps AI-related risks to corresponding governance guidelines within organizational contexts. Our framework, by contrast, is a *development-process* architecture: it addresses how governance is built across three functional layers over time, and specifically whether the social layer is developed in parallel with or sequentially after technical and ethical governance. The two models are complementary, but their purposes differ. Where Wirtz et al. address what risks organizations must govern, we address how governance across all actors and layers should be sequenced, and who bears responsibility at each stage of AI utilization.

### 3.2 Layer-based problems

The principal analytical utility of the layered governance model lies in its diagnostic capacity. By assigning AI-related issues to the layer in which their root causes are most clearly located, the framework enables more precise and actionable governance responses. A technical failure — such as a biased algorithm producing discriminatory outputs — calls for a technical-layer remedy. An ethical failure — such as a lack of transparency in consequential decision-making — calls for an ethical-layer response. A social failure — such as AI-driven labor displacement without adequate social protection mechanisms — demands social-layer governance. The accountability challenges associated with AI-generated content illustrate this diagnostic logic particularly clearly. Questions of intellectual property ownership, creative authorship, and legal opacity in AI outputs have already generated substantial debate in intellectual property law and the creative industries (Amankwah-Amoah et al., 2024; Tschider, 2021). These are not primarily technical failures — the systems generate content as designed — nor purely ethical failures in the sense of missing principles. They are social layer failures: the absence of enforceable legal frameworks governing ownership, liability, and transparency for AI-generated outputs at the point of public use. The layered diagnostic framework maps these debates to their correct governance layer and suggests the appropriate remedial action. Without this diagnostic clarity, governance interventions risk systematic misalignment: applying technical fixes to fundamentally social problems or expecting ethical principles alone to resolve what are essentially regulatory and legislative gaps. Table 1 presents an initial mapping of current AI governance challenges to their respective layers. This mapping is illustrative, not exhaustive: it provides a diagnostic starting point and should be extended through empirical research and regional adaptation, as noted in the Conclusion.

**Table 1:** Mapping of potential problems from AI using layered governance.

Technical layer	Ethical layer	Social layer
Reliability of AI model results Security of deployed AI systems Privacy and fairness of training data Interoperability failures between AI systems Absence of human override mechanisms in automated systems Environmental costs of large-scale model training	Social surveillance with AI Autonomous weapons powered by AI Financial crises brought about by AI Lack of informed consent in AI data collection Accountability gaps when AI causes harm Insufficient human-in-the-loop requirements in high-stakes AI Unequal distribution of AI’s environmental risks across actors	Job losses due to AI automation Social manipulation through AI algorithms Widening socioeconomic inequality as a result of AI Degradation of human qualities due to AI Erosion of public agency in AI-mediated decisions Community-level ecological impact of AI infrastructure

### 3.3 Governance development

**Figure 3:**
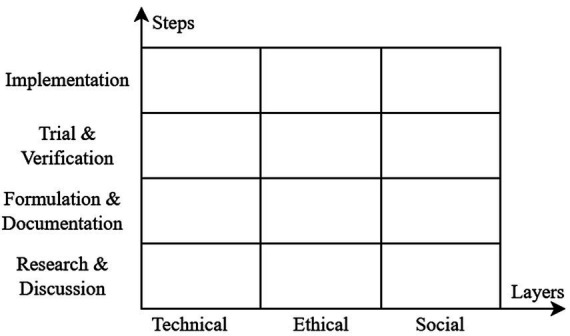
Governance development matrix.

Once governance challenges are mapped to their respective layers, a structured development process translates diagnosis into concrete action. We propose a two-dimensional governance development matrix (Figure 3) as a strategic blueprint. On one axis, the three governance layers; on the other, a four-step development process: (1) research and expert deliberation; (2) formal documentation and policy formulation; (3) piloting and empirical validation; and (4) full implementation and monitoring. These steps generalize the three-phase public decision-making framework proposed by de Fine Licht and de Fine Licht (2020) for AI-related policy contexts. Because the three layers must develop in parallel, progress across all matrix cells must be tracked simultaneously. A key implication of the society-oriented framework is that the social layer cells must not be left until last — as has historically been the case — but must be actively developed alongside the technical and ethical layers from the outset.

To operationalize the matrix, each step requires designated activities and actor responsibilities. Step 1 (research and expert deliberation) involves researchers, ethicists, and civil society representatives generating layer-specific evidence through technical risk assessments, ethical analysis, and social impact studies. Step 2 (documentation and policy formulation) involves government bodies and standards organizations translating deliberation outputs into governance instruments — both hard regulation (binding legislation and enforceable technical standards) and soft regulation (voluntary codes of practice and industry self-regulatory standards). This distinction matters: technical standards are more amenable to hard regulation, while social layer governance frequently requires the contextual flexibility of soft instruments. Step 3 (piloting and empirical validation) involves practitioners and government jointly testing instruments in real deployment contexts; for this conceptual framework, validation means structured expert panel review against documented governance failures and sector-specific case application. Step 4 (implementation and monitoring) is shared across all actors and is not terminal: it requires ongoing auditability mechanisms, incident reporting systems feeding failures back into Step 1, and periodic review cycles ensuring all three layers continue developing in parallel.

## 4 Multiple-actor framework

### 4.1 Four actors coordination

The governance development framework established in Section 3 addresses what needs to be governed. This section addresses who governs it and how coordination is sustained. The three governance layers function as filtering bridges between different stakeholders as AI knowledge passes through four successive stages before reaching end-users: (1) AI Innovation, where foundational research produces concepts, models, and frameworks; (2) platform implementation, where innovations are embedded into deployable systems and products; (3) practical application, where AI-powered products are deployed by professionals in specific domain contexts; and (4) public use, where society at large receives the benefits or bears the consequences of those applications.

**Figure 4:**
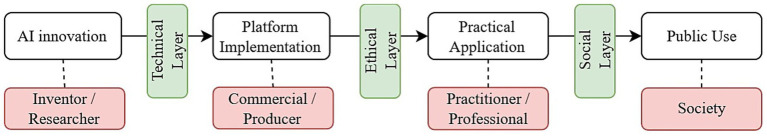
The three layers of governance become bridges for each stage in AI “lifecycle”.

**Figure 5:**
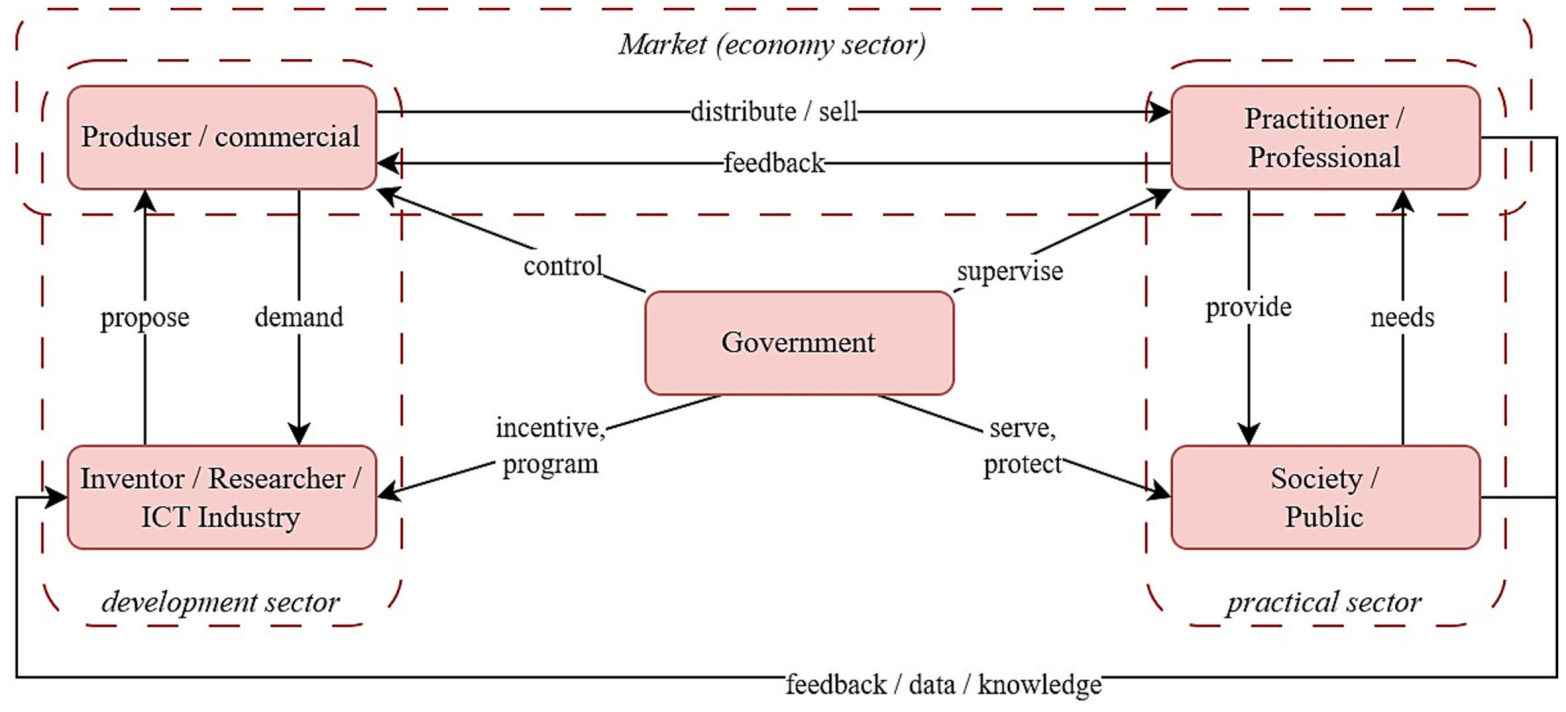
Relation diagram of 4-actors model.

Each stage corresponds to a distinct group of stakeholders and a distinct layer of governance. In Stage 1, researchers and inventors operate within the technical layer governance framework. In Stage 2, commercial producers translate innovations into products, governed primarily by ethical layer principles. In Stage 3, practitioners in specific fields apply AI-powered products, where both ethical and social layer governance must be operative. In Stage 4, social layer governance is the critical determinant of whether AI integration is beneficial, harmful, or ungoverned. As Figure 4 illustrates, each governance layer serves as a bridge between consecutive stages. In addition to these four primary actor groups, government plays a critical cross-cutting role as regulatory authority, standard-setter, and coordinator across all stages. As shown in Figure 5, government is positioned centrally in the actor relation diagram, maintaining connections to all sectors. This framework generalizes the healthcare-specific actor model proposed by Siadari et al. (2022) — which maps the relationships among AI researchers, technology developers, healthcare practitioners, and patients within a governance accountability structure — into a domain-agnostic 4-actors model applicable across sectors.

Mapping actors to stages is insufficient without specifying the governance obligations at each position. Researchers and innovators bear an obligation to publish transparency documentation including model risk assessments and known failure modes. Platform producers bear an obligation to implement socially responsive design standards as a condition of deployment — including usage transparency mechanisms, access controls, and pre-deployment societal impact assessments. Practitioners bear an obligation to exercise domain-specific ethical judgment and carry institutional accountability for AI-mediated outcomes in their professional context. Government bears an obligation to set minimum-standard social layer regulations, coordinate the federated board, and ensure governance obligations are not left to voluntary adoption. Making these obligations explicit converts the actors framework from a descriptive map into a governance architecture with defined accountability at each node.

### 4.2 Sectoral manifestation

**Figure 6:**
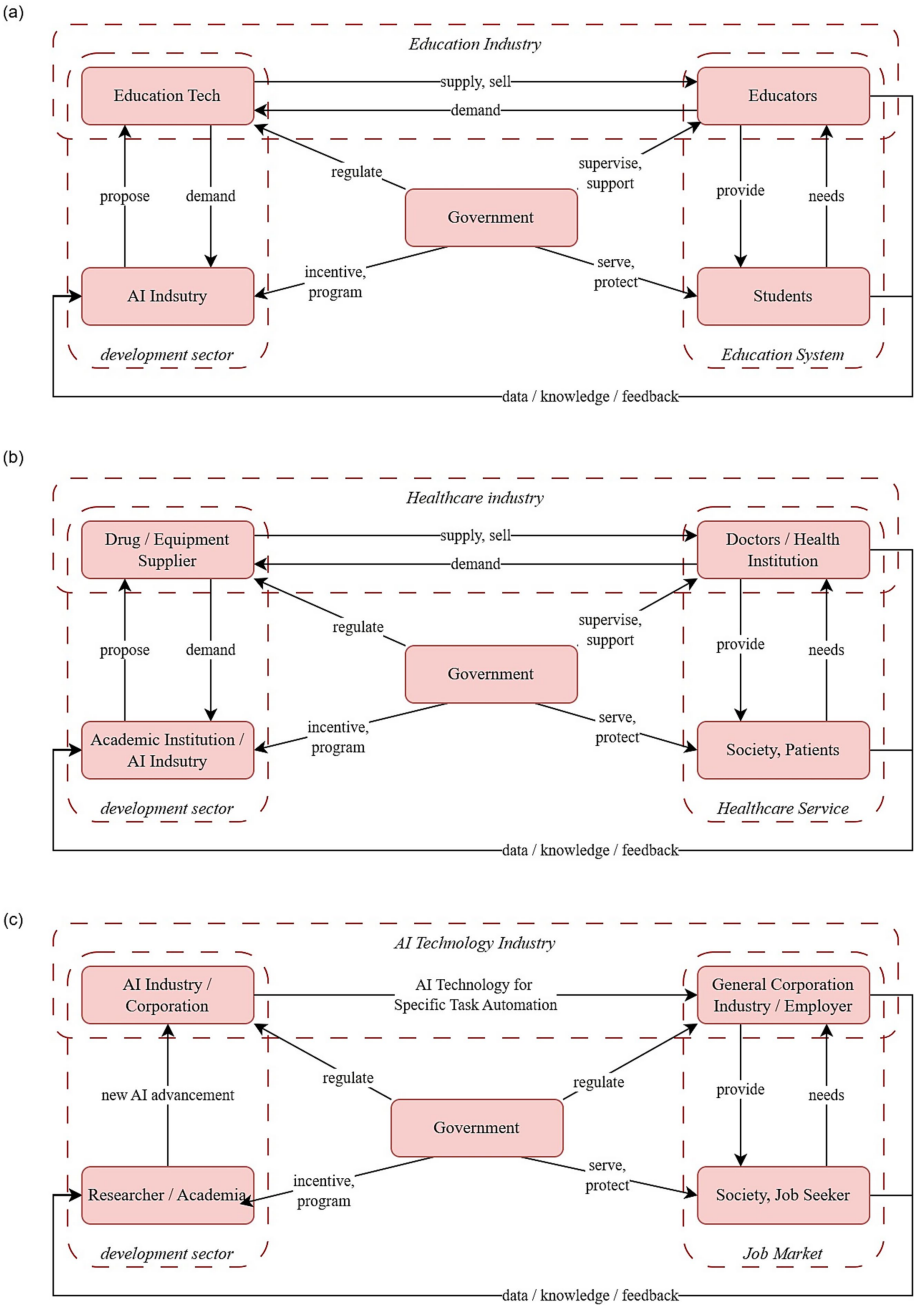
Diagram of 4-actors model in some examples of practical sector, as in **(a)** education, **(b)** healthcare, and **(c)** human resource.

The 4-actors model can be instantiated across any sector in which AI is deployed. Its analytical value lies not only in mapping who the relevant actors are, but in revealing where governance gaps exist: which actor relationships are under-governed, which layers are underdeveloped, and where the risk of ungoverned public impact is highest. We illustrate this with three sector-specific instantiations — education, healthcare, and human resources — which collectively demonstrate the framework’s versatility (Figures 6a–c).

In education, AI research tools are utilized by businesses running the education industry to create usable products for educational institutions. Students receive benefits indirectly through implementation by educators, who serve as supervisors ensuring the correct utilization of AI in the educational process (Guilherme, 2019). The primary governance gap is the absence of binding standards for AI products in contexts involving minors: access controls, transparency of AI-generated content, and educator oversight mechanisms remain largely unregulated at the platform level.

In healthcare, the framework is adapted from Siadari et al. (2022), with patients included as end-users. Good relations between doctors and patients represent the social layer of AI governance, as doctors not only provide knowledge services but also play the role of empathy (Nadin, 2020). The primary governance gap is an asymmetry of accountability: physicians bear full professional liability for AI-assisted decisions while platform producers bear limited liability for system failures that contribute to those decisions.

In human resource management, AI products adopted for task automation determine how corporations manage their human resources and affect the whole job market — a domain where the recent advancement of generative AI has raised significant labor replacement concerns (AbuMusab, 2023). The primary governance gap is the absence of auditability requirements: algorithmic hiring and performance management decisions routinely lack explainability mechanisms or appeal procedures for affected workers.

### 4.3 Three actors model: alternative cases

**Figure 7:**
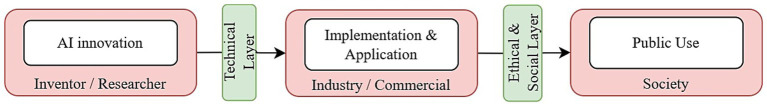
Diagram of 3-actors model as adjustment from 4-actors model for some cases of AI techs.

The 4-actors model assumes a professional intermediary between AI deployment and public use. This assumption holds in healthcare, education, and legal services, where AI is accessed through a qualified professional. However, many of the most societally consequential AI systems — including ChatGPT, social media recommendation algorithms, AI image generators, and autonomous consumer applications — bypass this intermediary entirely. By late 2024, ChatGPT alone had been reported to surpass 300 million weekly active users, a figure that has continued to grow rapidly, none of whom are mediated by a professional intermediary; similar patterns obtain across social media recommendation systems that reach billions of users directly (Weidinger et al., 2023). In these cases, the platform implementation and practical application stages merge: the producer creates a product the public uses directly, without a professional filter. This produces what we call the 3-actors model (Figure 7). Innovation, Platform/Application, and Public Use, with only a combined ethical-social governance layer mediating the transition. The 3-actors model is not a simplified edge case — it is a structurally distinct and more governance-vulnerable configuration that describes the dominant pattern of consumer AI deployment and demands specific policy attention.

In the 3-actors model, governance obligations that normally fall on a professional intermediary must be redistributed: upstream to platform producers through mandatory design standards, or laterally to the state through regulation. This has concrete implications. Platform producers in 3-actor configurations must bear obligations beyond those in professionally mediated contexts: (a) pre-deployment societal impact assessments across the likely user population; (b) usage transparency disclosures accessible to non-expert users; (c) age- and context-sensitive access controls; and (d) ongoing societal feedback mechanisms — structured channels through which public reception, emergent harms, and changing social norms are monitored and fed back into governance review. This last requirement is absent from all five major frameworks reviewed in Section 2.3. Without it, governance in 3-actor configurations is structurally static even as social conditions change dynamically.

## 5 Discussion

The frameworks proposed in Sections 3 and 4 provide analytical architecture for society-oriented AI governance. This section addresses three critical dimensions of their practical implementation: the structural question of how governance authority should be distributed, the substantive question of why the social layer demands particular urgency, and the scalar question of how global and national governance responsibilities should be divided. Each discussion closes with a concrete recommendation.

### 5.1 Centralized to federated

**Figure 8:**
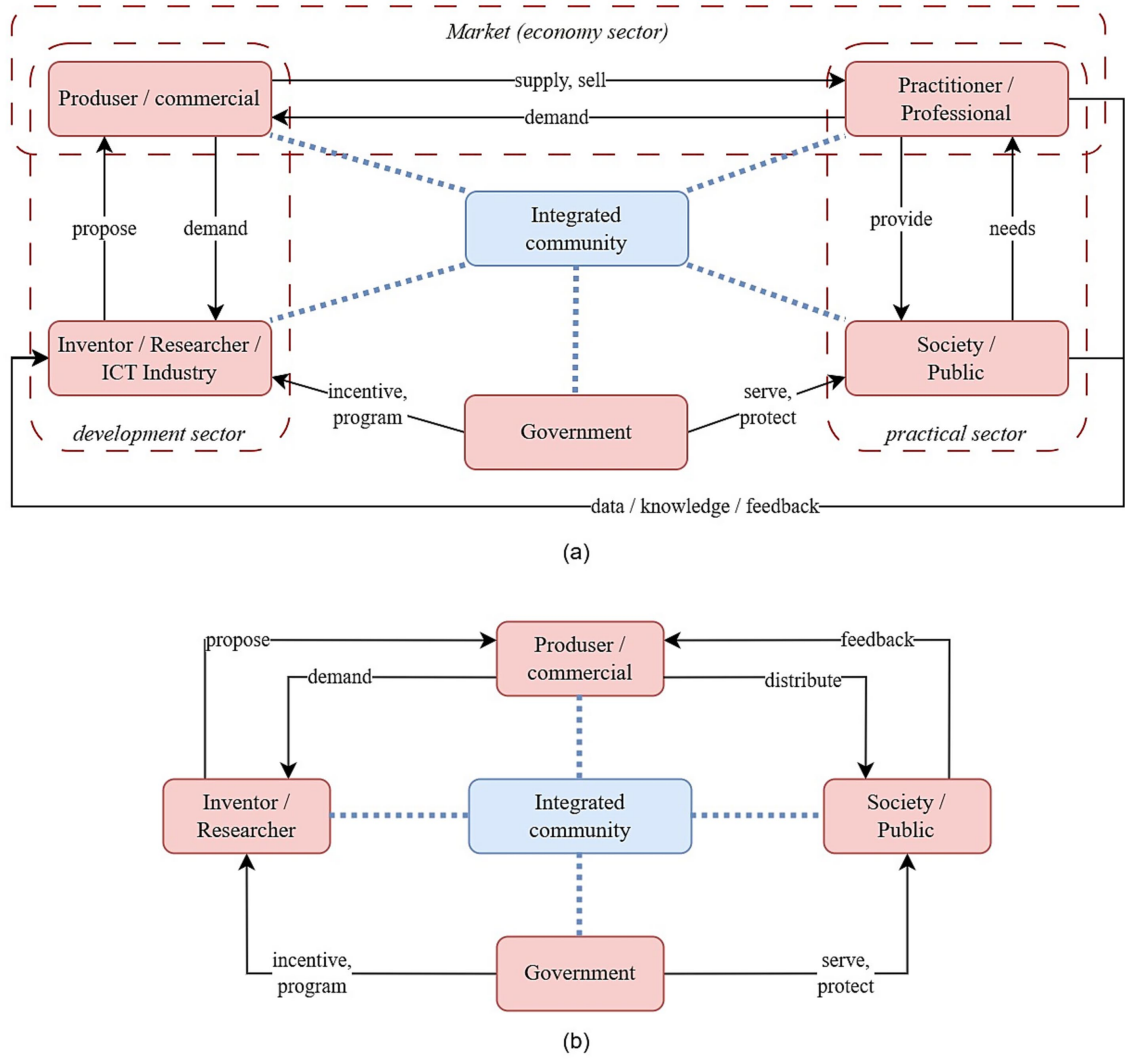
Diagram of federated paradigm version of both actor-based model governance framework. **(a)** Modified four-actors model; **(b)** Modified three-actors model.

In both the 4-actors and 3-actors models, government plays a pivotal cross-cutting role. In a centralized paradigm, government operates as the primary orchestrator, with authority flowing top-down to all other actors. While this model offers legal clarity, it is poorly suited to the pace and complexity of AI development: bureaucratic processes move slowly, regulatory capture is a persistent risk, and centralized authority creates bottlenecks that impede governance agility (Siadari et al., 2022). We therefore propose a federated governance model in which government assumes an equal position alongside other stakeholders within a self-regulatory, integrated coordination structure (Figure 8). This federated paradigm distributes governance responsibilities across actor groups while government retains its authority in matters of regulation and policy without acting as a central bottleneck. Akin to citizen-centric governance models in digital environments (Calzati and van Loenen, 2023), it promotes bottom-up agility while preserving accountability. While acknowledging the coordination challenges and power imbalance risks of federated models, we argue these are governance design challenges to be managed. *Recommendation: Establish a multi-stakeholder self-regulatory board with defined representation criteria from each actor group — AI research and innovation, platform producers, practitioner professional bodies, civil society and public interest organizations, and government regulatory agencies — tasked with coordinating governance development across all three layers simultaneously. To mitigate structural power asymmetries, representation criteria should include minimum guaranteed seats for civil society and public interest actors independent of resource capacity, with decision-making procedures that distinguish binding technical and regulatory instruments (requiring government authorization) from soft regulatory instruments (determined by cross-sector consensus). Governance of the board itself — including accountability mechanisms and conflict-of-interest protocols — should be subject to independent periodic review.*

To make this operational, the federated board requires representation from: (a) AI research and innovation; (b) platform producers; (c) practitioner professional bodies from major AI-intensive sectors; (d) civil society and public interest organizations; and (e) government regulatory agencies with binding authority. Decision-making authority should be differentiated: binding technical standards and hard regulation require government authorization; soft regulatory instruments — codes of practice, design guidance — are determined by cross-sector consensus. Hard regulation is appropriate where governance failures are severe or involve fundamental rights; soft regulation is appropriate where contextual variation makes uniform binding rules counterproductive. A federated board operating across both modes achieves legal clarity without sacrificing contextual adaptability.

A critical structural risk in any federated governance arrangement is regulatory capture — the process by which well-resourced actors systematically shape governance institutions to serve their interests at the expense of public accountability. In the AI context, this risk is acute: large platform producers command technical expertise, legal resources, and political influence that civil society organizations and smaller governments cannot match. The federated board design proposed here incorporates three specific countermeasures. First, minimum guaranteed representation seats for civil society and public interest actors, independent of resource capacity, prevent resource asymmetry from translating directly into representational exclusion. Second, the distinction between hard regulatory instruments — which require government authorization and are therefore not subject to industry consensus override — and soft regulatory instruments limits the scope within which resource-rich actors can dominate outcomes. Third, subjecting the board’s own governance to independent periodic review creates an external accountability mechanism that does not depend on the goodwill of the board’s most powerful members. We acknowledge that these measures mitigate rather than eliminate capture risk, and that empirical validation of their effectiveness in practice remains a necessary direction for future research.

### 5.2 Urgency of social layer

**Figure 9:**
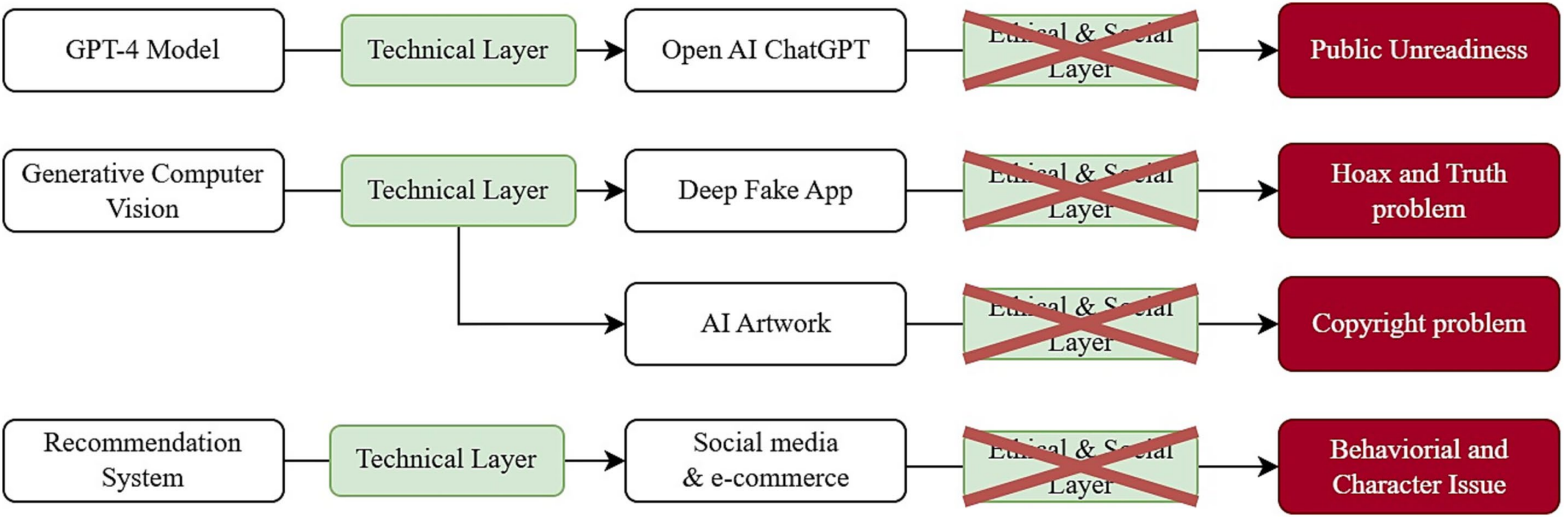
Illustration on dysfunction of ethical and social layer, which is one of the vital root causes of some issues.

Among the three governance layers, the social layer is simultaneously the most critical for society-oriented governance and the most underdeveloped in current practice. In the 4-actors model, the practitioner serves as a de facto social layer filter — someone with domain expertise, ethical training, and institutional accountability who mediates between AI systems and the public. This filter is what makes professional sectors comparatively well-governed: a doctor deploying an AI diagnostic tool is constrained by medical ethics, institutional review, and professional licensing in ways that a casual ChatGPT user is not. In the 3-actors model, this professional filter is absent. AI reaches the public directly, and the merged ethical-social governance layer must bear the full weight of mediating between deployment and use without sectoral specificity. As Figure 9 illustrates, this produces concrete failures: ChatGPT adoption without social readiness, deepfake proliferation without accountability mechanisms, AI art platforms without clear intellectual property governance. Each case shares the same structural root: an underdeveloped social layer. More precisely, each reflects the same governance mechanism failure: the absence of mandatory pre-deployment societal impact assessment, usage transparency disclosures, access controls calibrated to the actual user population, and incident reporting obligations — instruments that exist in analogous form in pharmaceutical safety monitoring, financial product disclosure, and media content regulation. *Recommendation: AI systems deployed in 3-actor configurations should be required to meet socially responsive design standards — including age-appropriate access controls, usage transparency mechanisms, and community impact assessments — as a condition of deployment* (Tigard et al., 2020)This approach resonates with the Ethics by Design tradition (d’Aquin et al., 2018; Dignum et al., 2018), which argues that ethical reflection should be embedded directly into the design and development process rather than applied retrospectively as a compliance check. Society-oriented governance extends this logic to the governance layer: just as Ethics by Design embeds ethical criteria into technical development, socially responsive design standards embed social layer requirements into platform deployment conditions. The TEAeM framework (Bhalla et al., 2025) similarly proposes integrating ethical assessment across technology development stages, which aligns with the parallel-layer architecture proposed here. This argument draws on a longer tradition: (Palm and Hansson, 2006) established the case for ethical technology assessment (eTA) as a systematic pre-deployment evaluation method — a precedent that reinforces the necessity of mandatory societal impact assessment as a social layer governance instrument for AI deployed in 3-actor configurations. Together, these perspectives reinforce the paper’s central argument that social layer governance cannot be an afterthought.

To make this recommendation operational, governments and governance bodies require concrete evaluation criteria. We propose three minimum indicators for assessing social layer adequacy. First, coverage: whether mandatory socially responsive design standards apply to all AI systems deployed in 3-actor configurations above a defined user-scale threshold. Second, accessibility: whether algorithmic transparency disclosures and user literacy mechanisms are demonstrably legible to non-expert users, assessed through structured public comprehension evaluation. Third, responsiveness: whether societal feedback mechanisms are actively monitored and whether documented incidents demonstrably feed back into governance review cycles within defined timeframes. Disagreements within the federated governance structure should be managed through a tiered resolution procedure: technical and factual disputes are referred to an independent expert panel; normative and policy disputes are escalated to the government regulatory representatives whose binding authority provides a decision backstop. This prevents deliberative deadlock while preserving the collaborative character of the federated model.

Specifically, these standards should require as a minimum: algorithmic transparency disclosures in user-legible language; mandatory user literacy onboarding for high-risk AI categories; community impact review requirements for population-scale platforms; and ongoing societal feedback mechanisms as specified in Section 4.3.

### 5.3 Scope division

**Figure 10:**
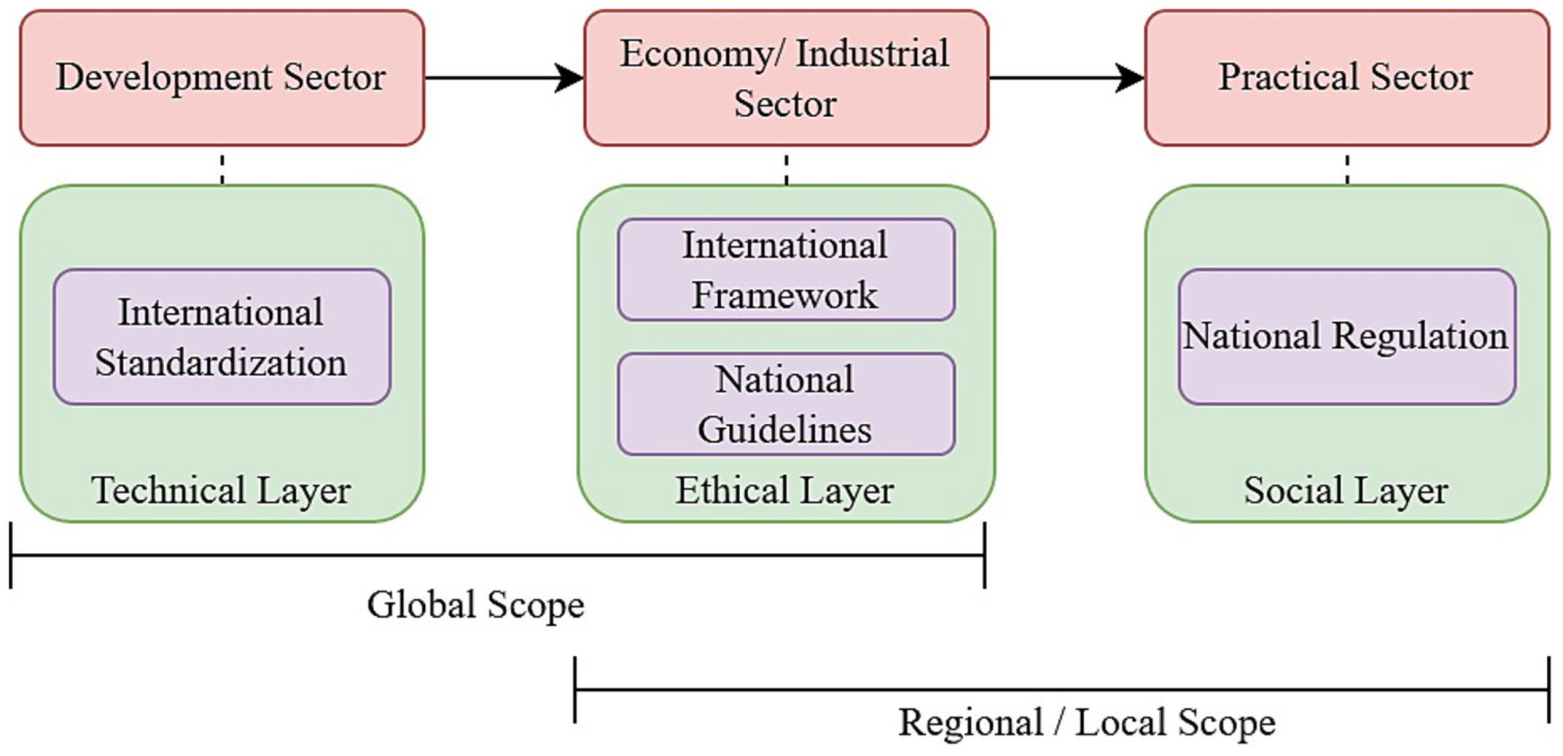
Division of scope for the layered model governance.

A recurring challenge in AI governance is the question of jurisdictional responsibility. The social layer is inherently local — shaped by cultural conditions, legal traditions, and community values that vary significantly across contexts — while the technical layer is inherently global, developed through international research and deployed across borders without geographic constraint (Gervais, 2023; Morley et al., 2023). Ethical governance must bridge these two scales. We propose a four-level scope hierarchy (Figure 10): (1) international standardization of AI systems (technical layer, global); (2) international ethical implementation frameworks (ethical layer, global); (3) national guidelines adapting international frameworks to domestic conditions (ethical layer, national); and (4) national/sub-national regulation governing AI access and use (social layer, national/local). The first two levels are relatively well-developed through OECD, UNESCO, and international standards bodies (Hagendorff, 2020). The NIST AI Risk Management Framework and ISO 23894 are the most operationally developed instruments at Levels 1 and 2, providing technically detailed and internationally applicable guidance. However, both are organization-facing: their mechanisms address how AI-deploying organizations manage risk, not how governments regulate societal access to AI. Levels 3 and 4 — national adaptation and national/sub-national social layer regulation — are outside their stated scope, and remain the most underdeveloped levels of the hierarchy. This paper’s primary contribution lies precisely at these two levels.

The third and fourth levels remain most underdeveloped and most critical for society-oriented governance. *Recommendation: National governments should develop minimum-standard social layer regulations that translate international ethical frameworks into enforceable, context-sensitive rules governing public access to and use of AI systems.* Establishing this scope hierarchy addresses a deep structural failure in current AI ethics discourse: the concentration of governance responsibility in the development sector, where ethics is frequently treated as a marketing signal rather than a binding commitment (Bankins and Formosa, 2023; Birkstedt et al., 2023).

A significant feasibility constraint warrants explicit acknowledgment. Several recommendations in this paper — mandatory pre-deployment impact assessments, federated multi-stakeholder boards, national-level social layer regulation — presuppose institutional capacity and governance infrastructure that may not be uniformly available, particularly in lower-income and developing country contexts. The scope hierarchy proposed in Section 5.3 partially addresses this by distinguishing global obligations (technical standardization, international ethical frameworks) from national ones (social layer regulation), recognizing that national implementation will vary. However, we acknowledge that Levels 3 and 4 of the hierarchy — precisely the levels identified as most underdeveloped and most critical — face the steepest implementation barriers in contexts with limited regulatory capacity. Two directions follow from this. First, international governance bodies operating at Levels 1 and 2 should incorporate capacity-building obligations alongside standard-setting functions. Second, the federated governance model should accommodate asymmetric participation arrangements that allow lower-capacity national actors to engage meaningfully without bearing the full institutional cost of compliance.

## 6 Conclusion

This paper has addressed a fundamental structural problem in AI governance: the systematic underrepresentation of society — as the ultimate recipient of AI’s consequences — in the design of governance frameworks. Using the rapid and ungoverned public adoption of ChatGPT as a paradigmatic case, we have demonstrated that governance frameworks anchored in the development sector are structurally insufficient to address harms that materialize at the point of public use.

The governance development framework modifies Gasser and Almeida (2017) by replacing its sequential hierarchy with a parallel, dynamic architecture in which the technical, ethical, and social layers develop simultaneously and in continuous mutual interaction. The multiple-actor framework maps stakeholders across the four stages of AI utilization and proposes a federated coordination structure that distributes governance responsibility without eliminating accountability. The 3-actors model is identified as a structurally distinct and more governance-vulnerable configuration describing the dominant pattern of consumer AI deployment. Three cross-cutting findings emerge: first, the social layer is the most critical and most underdeveloped layer in current AI governance; second, a federated governance paradigm better serves the agility required by society-oriented governance than centralized models; and third, governance responsibility must be explicitly distributed across global, national, and local scopes.

Several limitations merit acknowledgment. Both frameworks are deliberately abstract — analytical lenses and strategic architectures, not implementation protocols. The complexity of real-world governance environments will introduce frictions that the frameworks do not fully address. Power asymmetries between actors may systematically distort federated governance in practice. The problem mapping in Table 1 is illustrative, not exhaustive. Three validation directions are proposed. First, structured expert panel evaluation: the framework’s diagnostic categories and governance mechanism proposals should be reviewed by a cross-disciplinary panel of AI governance scholars and regulatory practitioners from multiple jurisdictions. Second, sector-specific case application: applying the actors model, development matrix, and scope hierarchy to a specific national governance failure to test diagnostic utility and derive concrete design recommendations. Third, comparative national analysis: examining how existing national AI governance systems map onto the four-level scope hierarchy would identify where Levels 3 and 4 are most underdeveloped and where society-oriented reorientation is most urgently needed. These directions convert the paper’s limitations into a tractable research agenda.

These validation directions also imply criteria for disconfirmation. The parallel-layer model would be challenged by systematic evidence that sequential governance development produces comparably effective societal outcomes in openly accessible AI contexts — specifically, evidence that social layer governance can be adequately developed after, rather than alongside, technical and ethical layer governance without producing observable harm gaps. The 3-actors model would be challenged by evidence that professional intermediaries are present and functionally operative in the consumer AI deployment contexts identified here, or that platform producers in 3-actor configurations already bear and enforce governance obligations equivalent to those proposed. We welcome such evidence as a productive contribution to the research agenda.
